# Gas Sensor for Efficient Acetone Detection and Application Based on Au-Modified ZnO Porous Nanofoam

**DOI:** 10.3390/s24248100

**Published:** 2024-12-19

**Authors:** Zhenchao Sun, Shanfu Sun, Xidong Hao, Yinglin Wang, Caili Gong, Pengfei Cheng

**Affiliations:** 1 School of Aerospace Science and Technology, Xidian University, Xi’an 710126, China; 13561113629@163.com (Z.S.); haoxidong@xidian.edu.cn (X.H.); wangyl@xidian.edu.cn (Y.W.); 2 School of Electronic Information Engineering, Inner Mongolia University, Hohhot 010021, China; eegcl@imu.edu.cn

**Keywords:** acetone gas sensor, metal oxide-semiconductor, porous nanostructure, Au/ZnO, portable device

## Abstract

Toxic acetone gas emissions and leakage are a potential threat to the environment and human health. Gas sensors founded on metal oxide semiconductors (MOS) have become an effective strategy for toxic gas detection with their mature process. In the present work, an efficient acetone gas sensor based on Au-modified ZnO porous nanofoam (Au/ZnO) is synthesized by polyvinylpyrrolidone-blowing followed by a calcination method. XRD and XPS spectra were utilized to investigate its structure, while SEM and TEM characterized its morphology. The gas sensitivity of the Au/ZnO sensors was investigated in a static test system. The results reveal that the gas-sensitive performance of porous ZnO toward the acetone can be enhanced by adjusting the loading ratio of noble Au nanoparticles. Specifically, the Au/ZnO sensor prepared by the Au loading ratio of 3.0% (Au/ZnO-3.0%) achieved a 100 ppm acetone gas response of 20.02 at the optimum working temperature of 275 °C. Additionally, a portable electronic device used a STM32 primary control chip to integrate the Au/ZnO-3.0% gas sensor with other modules to achieve the function of detecting and alarming toxic acetone gas. This work is of great significance for efficiently detecting and reducing acetone emissions.

## 1. Introduction

Volatile organic compounds (VOCs) such as acetone, xylene, nitrogen dioxide, and sulfur oxide are prevalent contaminants in the air, which are recognized as being poisonous and potentially cancer-causing to humans [1,2,3]. On the other hand, these VOCs have a wide range of roles in the chemical industry [4]. Therefore, there is a potentially serious threat to human health when these VOCs leak during transportation and application. In addition, more serious consequences such as explosions, leading to casualties and property damage, may also occur [5,6]. As a typical VOC, acetone has the potential to harm the kidneys, liver, brain, and nervous system in humans [7]. A convinced time-weighted average (TWA) exposure limit of acetone is 35 parts per million (ppm) [8]. As a consequence, developing a swift and real-time detection technology for acetone is crucial for safeguarding human health.

Gas sensors that function based on the mechanism of resistance variation while the sensitive materials react with the gases in contact are an ideal acetone gas detection method [9]. Over the last few years, metal oxide semiconductors (MOS) have garnered considerable attention, due to their established manufacturing processes, chemical stability, and cost-effectiveness [10]. Gas sensors fabricated by Fe_2_O_3_ [11], SnO_2_ [12,13], NiO [14], and ZnO [15,16] have been confirmed to show sensitive responses to acetone. As a representative n-type semiconductor, ZnO has obtained extensive attention in the field of gas sensors because of its non-toxicity, exceptional thermal stability, and high electron mobility [17,18,19]. ZnO possesses the capability to detect both oxidizing and reducing gases across a broad range [20]. Nevertheless, its selectivity and response for gas detection need to be further enhanced in applications. Increasing surface areas of ZnO by morphology engineering, such as nanowires [21,22], nanosheets [23,24], and nanospheres [25,26] can provide more active sites and thus enhance its reaction efficiency. For instance, Sun et al. synthesized peony-shaped ZnO via a hydrothermal approach. The gas-sensitive response of the prepared ZnO to 100 ppm ethanol at an operating temperature of 350 °C attained a value of 17.4 [27]. Choi et al. synthesized porous ZnO nanosheets through the solvothermal method [24]. As a gas sensor, it exhibited a response of 74.68 to 10 ppm NO_2_ at 200 °C. The porous foam structure exhibits a high surface and volume ratio [28]. This characteristic endows it with the capacity to generate copious active sites and oxygen vacancies, which in turn favors gas adsorption and desorption processes, promotes molecular activation, and expedites gas diffusion and response [29,30]. Consequently, it has emerged as an ideal sensing material.

Noble metals modification on MOS is an efficacious approach to improving their gas responses, due to the effects of chemical and electronic sensitization [31]. Because of the high electronegativity and catalytic activity of the noble metal, it can adsorb and stabilize an increased number of oxygen atoms on its surface. These adsorbed oxygen atoms not only improve the interaction of the material with target gas molecules but also promote the activation process of the gas molecules, thus improving the gas-sensitive properties [32]. As an example, Wang et al. prepared Au-loaded ZnO flowers by a thermal decomposition in organic solvents, which showed high acetone-sensing performance [33]. Zhou et al. synthesized sea urchin-like Pd doping W_18_O_49_ nanocomposites [34]. As a gas sensor, its response value reached 32.0 to 50 ppm of H_2_ at a temperature of 100 °C. Yu et al. prepared core-shelled Au@In_2_O_3_ nanoparticles by a hydrothermal method and investigated their sensing properties for H_2_ [35]. It was found that the as-prepared Au@In_2_O_3_ sensors showed a 4-fold increase in response to 100 ppm H_2_ at the most effective working temperature of 300 °C compared to the bare In_2_O_3_ nanoparticles.

Herein, an efficient acetone gas sensor based on Au-modified ZnO porous nanofoam (Au/ZnO) is designed. The sensitive materials of Au/ZnO were synthesized through polyvinylpyrrolidone (PVP)-blowing followed by a calcination method. Its porous nanofoam structure is advantageous to exposing added reaction-active sites. Au nanoparticle modification increased the depth of the electron depletion region by transferring electrons from ZnO to Au. This synergistic effect enabled the optimal ZnO with 3.0% Au loading (Au/ZnO-3.0%) to exhibit an efficient acetone-sensitive performance, which achieved a gas response of 20.02 to 100 ppm acetone at the optimum working temperature of 275 °C. Additionally, a portable electronic device integrating with the Au/ZnO-3.0% gas sensor achieved the function of detecting and alarming toxic acetone gas. This work provides an efficient strategy for detecting organic toxic gases.

## 2. Experimental Section

### 2.1. Materials

Zinc nitrate hexahydrate (Zn(NO_3_)_2_·6H_2_O, 99%), polyvinylpyrrolidone (PVP, Mw: 55,000), ammonia water (NH_3_·H_2_O, 25–28%), gold chloride trihydrate (HAuC_l4_·3H_2_O, ≥99.9%), and acetone (≥99.5%) were obtained from Aladdin Chemical Co., Ltd. (Shanghai, China) and utilized directly without other treatment.

### 2.2. Synthesis of the Au-Modified ZnO Porous Nanofoam

The bare ZnO porous nanofoam was firstly synthesized via PVP-blowing, followed by a calcination method, as we previously reported [36]. In brief, 2.694 g of Zn(NO_3_)_2_·6H_2_O and 1.308 g of PVP were mixed in 15 mL of anhydrous ethanol, then 5 mL of NH_3_·H_2_O was dropped in under magnetic stirring until the solution was clarified. The obtained white precipitate was subjected to drying in an oven at a temperature of 60 °C for a duration of 12 h, followed by heating up to 500 °C in a box furnace at a temperature increase of 2 °C/min for 1.5 h to prepare the bare ZnO. Afterward, 10 mL of solution containing various mass ratios (0, 0.5, 1.0, 3.0, and 5.0%) of HAuCl_4_·3H_2_O was dropped into the as-prepared mixture containing 0.2 mmol ZnO in 10 mL solution under constant magnetic stirring for 6 h. The mixture was dried in air and subsequently calcinated at 500 °C for 1.5 h at a temperature elevation rate of 2.0 °C/min. Finally, the noble Au-modified ZnO porous nanofoams were synthesized. The obtained samples were defined as ZnO, Au/ZnO-0.5%, Au/ZnO-1.0%, Au/ZnO-3.0%, and Au/ZnO-5.0%, respectively.

### 2.3. Materials Characterization

X-ray diffraction (XRD, Rigaku D/Max 2550, Rigaku Corporation, Tokyo, Japan) with Cu-Kα radiation was carried out to analyze the crystal and phase structure of the sensitive materials. Field emission scanning electron microscopy (SEM, JEOL JSM-5610LV, JEOL Ltd., Tokyo, Japan) and transmission electron microscopy (TEM, FEI TecnaiG2S-Twin microscopes, FEI Company, Hillsboro, America) were employed to investigate their morphology and nanostructure. An X-ray photoelectron spectrometer (XPS, Thermo Scientific K-Alpha XPS, Themo Fisher, Massachusetts, America) with Al-Kαsource (1486.71 eV) was utilized to investigate the surface elemental composition and valences.

### 2.4. Fabrication and Measurement of Gas Sensor

The gas sensor employed in the work is a representative side-heated resistive type. The fabrication process of gas sensors comprises the following steps. Initially, 0.01 g of the as-prepared sensitive material was added and ground into a slurry by incorporating 0.2 mL of deionized water. Thereafter, the slurry was evenly applied to the surface of the ceramic tube and then calcined in a muffle furnace at 350 °C for 2 h, ensuring that the sensitive material adhered tightly to the ceramic tube. After this, a Ni–Cr alloy resistance wire was placed inside the calcined ceramic tube and soldered to the hexagonal base. To guarantee stability during testing, the sensors required additional aging in the air at 200 °C for 3 d. The active layer thickness of sensitive materials coated on ceramic tube is ca. 30 μm (Figure S1).

The gas-sensitive properties of the sensors were examined within the laboratory environment. The test system is composed of four components. A three-output DC power supply (OWON-ODP3033, Fujian Lilliput Optoelectronics Technology Co., Ltd., Fujian, China) is utilized for the control of the operating temperature. Two airtight containers with a volume of 1.0 L each are incorporated, wherein one can be filled with raw air and the other with target gas. A multimeter (Fluke 8846a, Fluke Corporation, Washington, America) is employed to record the real-time resistances of the sensors. A computer is integrated to display and store the test data. The as-fabricated sensors were positioned in a 1.0 L airtight container and the target gas was injected into the container by a micro syringe. The specific concentration of the target gas is determined through the static liquid–gas distribution method, and this concentration can be calculated using a specific formula [37]: C = 1000 × [(22.4 × ϕ × ρ × V_1_)/(M × V_2_)], where C (ppm) is the gas concentration, ϕ is the gas volume fraction, ρ (g/mL) is the liquid density, V_1_ (μL) is the liquid volume, V_2_ (L) is the chamber volume, and M (g/mol) is the liquid molecular weight. For n-type sensors, their response to the target gas can be expressed as S = R_a_/R_g_, where R_a_ and R_g_ represent the stable resistance value in air and the stable resistance value in the target gas, respectively.

## 3. Results and Discussion

### 3.1. Morphology and Structure of Sensitive Materials

**Figure 1:**
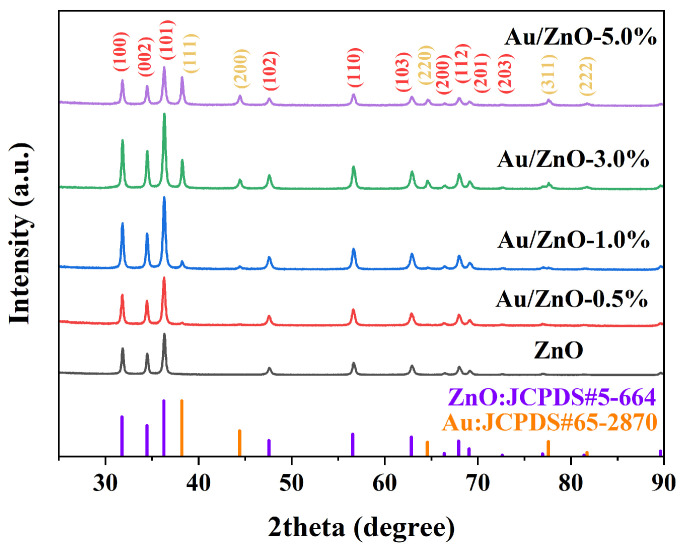
XRD patterns of the bare ZnO, Au/ZnO-0.5%, Au/ZnO-1.0%, Au/ZnO-3.0%, and Au/ZnO-5.0% samples.

The structures of the as-prepared sensitive materials were first investigated by XRD measurement. As shown in Figure 1, the diffraction peaks of the bare ZnO at 31.75°, 34.44°, 36.25°, 47.54°, 56.55°, 62.87°, 66.39°, 67.92°, 69.06°, and 89.63° correspond to the (100), (002), (101), (102), (110), (103), (200), (112), (201), and (203) crystallographic planes of wurtzite hexagonal (JCPDS No. 5-664), respectively, and no other redundant characteristic peaks occurred. After the Au nanoparticle modification, the extra diffraction peaks located at 38.19°, 44.39°, 64.58°, 77.57°, and 81.72° index to the (111), (200), (220), (311), and (222) crystal planes of face-centered cubic Au (JCPDS No.65-287), respectively. In addition, the intensities of these XRD peaks are enhanced with the increase of the amount of Au loading in the Au/ZnO-0.5%, Au/ZnO-1.0%, Au/ZnO-3.0%, and Au/ZnO-5.0% materials. The Scherrer equation (D = kλ/(βcosθ)) was employed to calculate particle sizes of the as-obtained Au/ZnO-3.0%, where k = 0.89, λ = 0.15406 nm, β represents full width at half maximum (radian), and θ = 38.22°. Accordingly, the particle size of Au loaded on porous Au/ZnO-3.0% was calculated to be 45.8 nm. XRD analyses demonstrated that the sensitive materials of Au-modified ZnO were successfully prepared.

**Figure 2:**
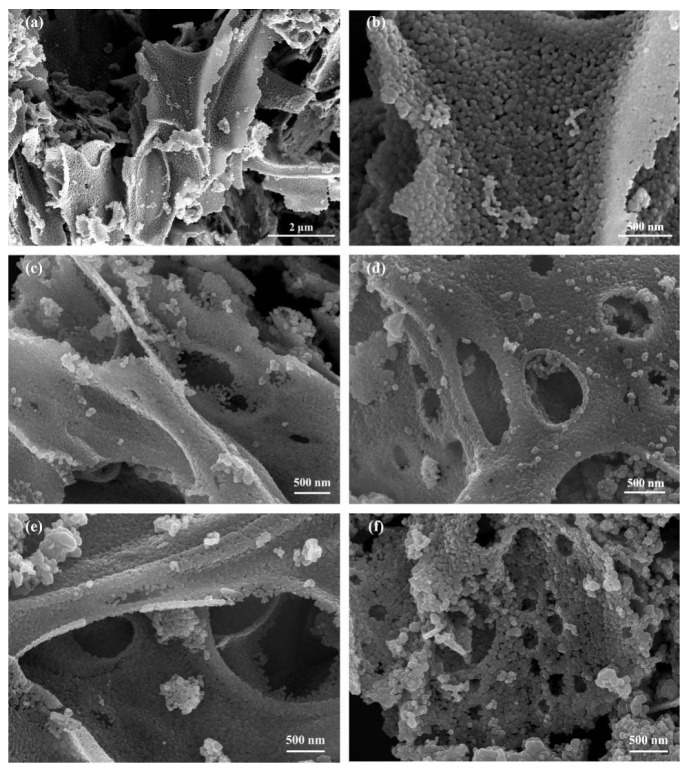
SEM images of the as-synthesized sensitive materials: (**a**,**b**) bare ZnO; (**c**) Au/ZnO-0.5%; (**d**) Au/ZnO-1.0%; (**e**) Au/ZnO-3.0%; and (**f**) Au/ZnO-5.0%.

The morphologies of as-prepared Au/ZnO sensitive materials are characterized by the SEM and TEM measurements. As can be seen in Figure 2a, the bare ZnO synthesized by PVP-blowing followed by a calcination method displays a porous, three-dimensional, interconnected nanofoam structure. Further characterization reveals that the two-dimensional nanosheets consist of numerous self-assembled nanoparticles (Figure 2b). In addition, its overall porous network nanostructure is well-retained after the Au nanoparticles have been modified in all Au/ZnO samples (Figure 2c,d). On the other hand, the Au nanoparticles are hard to distinguish from the SEM images.

**Figure 3:**
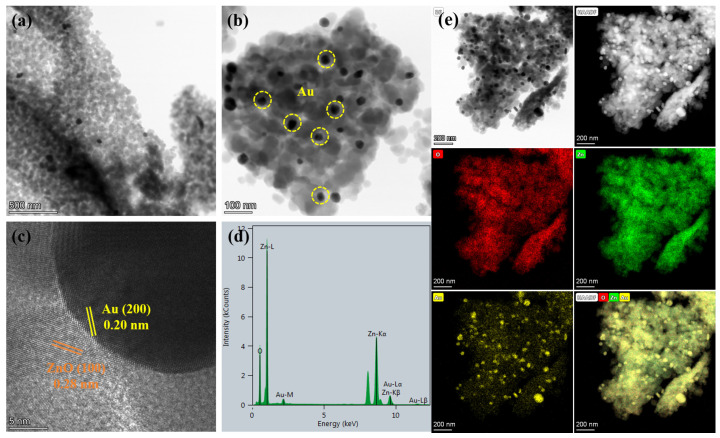
(**a**,**b**) TEM images; (**c**) HRTEM image; (**d**) EDX spectrum; and (**e**) high-angle annular dark-field TEM (HAADF-TEM) and EDX elements mappings of the Au/ZnO-3.0% sample.

Fortunately, as seen in the TEM images of Au/ZnO-3.0% shown in Figure 3a,b, the Au nanoparticles with sizes of 20–50 nm interspersed on the porous ZnO networks can be observed. The HRTEM image in Figure 3c presents well-resolved lattice fringes featuring spacings of 0.28 nm and 0.20 nm, corresponding to the (100) plane of wurtzite hexagonal ZnO and (200) plane of face-centered cubic Au, respectively. Moreover, the energy-dispersive X-ray (EDX) spectrum demonstrates the simultaneous existence of Au, Zn, and O elements (Figure 3d). HAADF-TEM image and EDX elements mappings further reveal the uniform distribution of the three elements (Figure 3e). The morphological characterization combined with the XRD results confirm that the Au nanoparticles modified ZnO with porous networks were obtained. The porous nanostructure helps to improve the adsorption capacity of gas molecules and enhance the sensitivity of gas detection [38]. In addition, the loading of precious Au can augment the interaction sites between ZnO and the target gas, consequently speeding up the reaction rate and elevating the sensitivity to the target gas [39,40].

**Figure 4:**
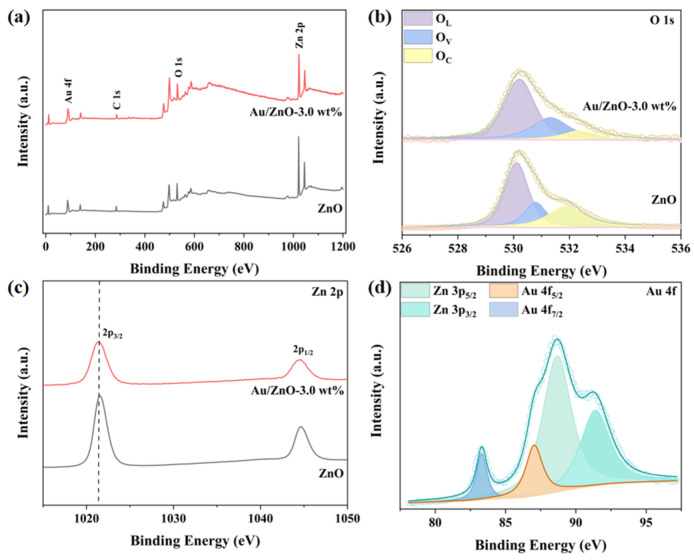
XPS spectra of Au/ZnO-3.0% and pure ZnO sensitive material: (**a**) XPS survey spectrum; (**b**) O 1s; (**c**) Zn 2P; and (**d**) Au 4f.

XPS measurement, conducted to investigate the oxidation state of the surface elements on Au/ZnO-3.0%. XPS survey spectrum (Figure 4a) further demonstrates the coexistence of the Au, O, and Zn elements on its surface. The O 1s spectrum is resolved into three distinct peaks, corresponding to lattice oxygen (O_L_) at 530.2 eV, oxygen vacancies (O_V_) at 531.4 eV, and adsorbed oxygen (O_C_) at 532.3 eV (Figure 4b) [36]. Notably, the O_V_ peak is particularly significant, as it interacts with VOC gases, thereby influencing the sensing performance [41]. A higher O_V_ content of Au/ZnO-3.0% (28.46%) than pure ZnO (21.01) reveals that Au nanoparticle modification facilitates the gas response. Two typical peaks of the Zn 2p spectrum located at the 1021.8 eV (2p_3/2_) and 1044.9 eV (2p_1/2_) reveal that it presents the Zn^2+^ (Figure 4c) [36]. A 0.1 eV blue shift of Au/ZnO-3.0% compared to pure ZnO further proves that more O_V_ was generated in the former and the electrons moved toward Zn^2+^ [36]. In the Au 4 f spectrum, two peaks located at 83.5 eV and 87.2 eV can be attributed to Au 4f_7/2_ and Au 4f_5/2_, respectively, suggesting that Au manifests the zero valence (Figure 4d) [42,43]. Another phenomenon should be highlighted, that the additional peaks at 88.3 eV and 91.7 eV are the result of the overlap of Zn 3p. The XPS results further verified that the noble Au nanoparticles are loaded onto the porous ZnO matrix in the Au/ZnO-3.0% sample.

### 3.2. Gas Sensing Performance

**Figure 5:**
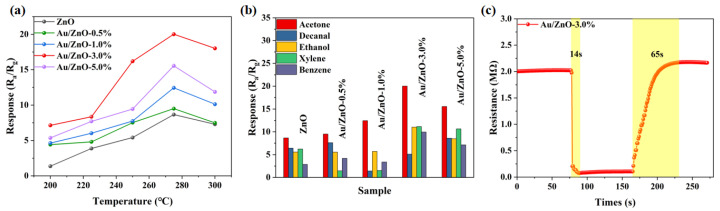
The gas-sensing properties of ZnO, Au/ZnO-0.5%, Au/ZnO-1.0%, Au/ZnO-3.0%, and Au/ZnO-5.0% to 100 ppm acetone: (**a**) Response to 100 ppm acetone at 200–300 °C; (**b**) responses to 100 ppm various target gases at 275 °C; and (**c**) response and recovery time of the Au/ZnO-3.0% sensor to 100 ppm acetone at 275 °C.

Subsequently, the acetone gas-sensing performance of Au-modified ZnO porous nanofoam was explored. The responses of MOS gas sensors are susceptible to temperature variations [44,45]. It is necessary to test the performance of toxic gas sensors at different operating temperatures to ascertain the optimum working temperature. Therefore, the as-prepared sensors of ZnO, Au/ZnO-0.5%, Au/ZnO-1.0%, Au/ZnO-3.0%, and Au/ZnO-5.0% were utilized to test acetone gas-sensing response with a concentration of 100 ppm at temperatures ranging from 200 to 300 °C. The resistance in the air (R_a_) of the sensors was detected (Figure S2). Their resistances decreased with increasing temperature in the range of 200–300 °C, due to the interaction of electronic thermal excitation and surface reaction processes [36]. As displayed in Figure 5a, at the initial stage, the response of all sensors rose as the temperature increased, peaking at 275 °C, and subsequently exhibited a downward trend as the temperature continued to rise. Therefore, gas sensors can efficiently sense acetone gas at 275 °C. The reason for this can be ascribed to the adsorption capacity of sensitive materials for acetone gas molecules enhanced with temperature upgrades below 275 °C, whereas a fast molecule desorption reaction will occur when the operating temperature is beyond 275 °C, thus decreasing the gas response [46]. Another reason is that the Au nanoparticle modification efficiently boosts the porous ZnO network’s sensitive response toward acetone gas and also presents an initial rise followed by a decline, while the Au loading amounts increase from 0.5% to 5.0%. This is primarily attributed to the fact that, upon the addition of a small quantity of Au, the specific surface area of the sensitive material is increased, thereby enhancing the adsorption capacity of the sensitive material. However, when the Au doping amount exceeds 3.0%, although the surface catalytic activity of the sensitive body is augmented, the desorption process of acetone gas by Au is also intensified. This leads to a reduction in the adsorbed amount of acetone and precludes further improvement in the performance of the gas sensor [37]. In particular, the Au/ZnO-3.0% sensor exhibits a peak response value reaching 20.02 at the optimum working temperature of 275 °C. Its performance is superior and comparable to most of the recently reported literature (Table S1). In addition, other toxic gases may affect the detection of acetone in daily applications. Therefore, the gas selective responses toward 100 ppm of several VOCs, such as acetone, decanal, ethanol, xylene, and benzene on the fabricated sensors at the optimal operating temperature of 275 °C were analyzed. As shown in Figure 5b, compared to other gas sensors, the Au/ZnO-3% shows a superior response to these gases and an exclusive response to acetone, revealing that the Au/ZnO-3% gas sensor possesses favorable selectivity. The gas-sensing characteristics of toxic gas sensors are manifested through changes in resistance values, and response and recovery time also constitutes crucial parameters for the assessment of the gas sensor performance [47,48]. Therefore, the resistance change curve of the Au/ZnO-3.0% gas sensor to 100 ppm acetone at 275 °C was tested (Figure 5c and Figure S3). The Au/ZnO-3.0% gas sensing material is an n-type semiconductor metal oxide, whereas acetone is a reducing gas [18]. When the Au/ZnO-3.0% gas sensor contacts with acetone gas, its resistance value will rapidly decrease. Nevertheless, when the gas sensor leaves the acetone gas and contacts with air again, the resistance value rapidly increases and can return to its initial value after some time. It was calculated that the response and recovery time of the Au/ZnO-3.0% gas sensor to acetone gas are 14.0 s and 65.0 s, respectively.

**Figure 6:**
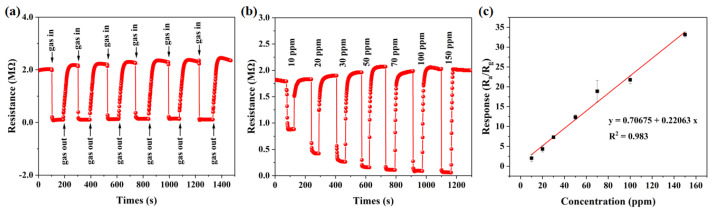
(**a**) Reversible cycles of Au/ZnO−3.0% to 100 ppm acetone at 275 °C; (**b**) the concentration gradient dynamic response curve of Au/ZnO−3.0% to 10−150 ppm acetone at 275 °C; and (**c**) the corresponding linear fit curve.

The cyclic repeatability of gas sensors under complex gas conditions is another important criterion for applications [49]. Figure 6a illustrates six cycles of response and recovery for the Au/ZnO-3.0% gas sensor when exposed to 100 ppm of acetone gas at a temperature of 275 °C. In the operating processes, the response of the Au/ZnO-3.0% gas sensor changed little and showed fast response and recovery speeds. This observation demonstrates that the gas sensor has excellent repeatability. Figure 6b shows the dynamic response-recovery cycle curve of acetone gas sensors at the optimal operating temperature of 275 °C, illustrating their performance across various gas concentrations ranging from 10 ppm to 150 ppm. It can be observed that the response of the sensor escalates progressively as the concentration of acetone increases. The sensor response value remains at 2.02 when the minimum concentration of acetone is 10 ppm. However, its response value can reach 33.16 at a high acetone concentration of 150 ppm, suggesting that the Au/ZnO-3.0% gas sensor possesses a wide concentration detection range for acetone. Figure 6c depicts the linear fitting curve that correlates the acetone concentration with the response of the gas sensor. The error bars in the figure are the errors obtained after repeating the experiment three times. The fitting curve is formulated as y = 0.22063 × x + 0.70675, with an R^2^ value of 0.983. Here, x denotes the concentration of acetone gas, y signifies the response value, and R^2^ reflects the strength of the fitted relationship. This equation demonstrates a positive and linear correlation between y and x and the value of R^2^ is close to 1.0, which means that the Au/ZnO-3.0% gas sensor fitting conforms to the linear law [50].

The mechanism of Au-modified porous ZnO networks for acetone detection was further analyzed. Combined with our previous report [37], ZnO is a representative n-type semiconductor sensing process, which involves the surface reaction between the adsorbed oxygen and acetone gas, thus leading to changes in resistance [32]. The O_2_ molecules initially adsorb onto the ZnO surface and capture electrons from its conduction band, leading to the formation of various oxygen species (O^2−^, O^−^, and O^2−^) when exposed to air. Therefore, a depletion layer of electrons forms on the ZnO surface, resulting in the resistance increase. When the ZnO sensor is utilized to detect acetone, the adsorbed oxygen species will undergo a reaction with acetone molecules, freeing the captured electrons and allowing them to return to the conduction band of ZnO. As a consequence, its resistance would be decreased. As shown in Figure 5, noble Au modification further enhanced the gas-sensitive responses of porous ZnO. On the one hand, an in-built electric field is constituted at the Au/ZnO-3.0% interface due to the higher work function of Au (5.1 eV) than that of ZnO [51]. It will drive electrons to transfer from ZnO to Au and leave a portion of holes on the ZnO surface, thus increasing the thickness of the electron depletion layer. On the other hand, Au nanoparticles on ZnO can be used as active sites to adsorb and decompose oxygen and form more negative oxygen ions because of the spillover effect of noble metals [52]. This will accelerate the interaction between acetone gas molecules and adsorbed oxygen species. Hence, the acetone gas sensing performances of porous ZnO networks are improved after Au nanoparticle modification.

## 4. Acetone-Detecting Device

**Figure 7:**
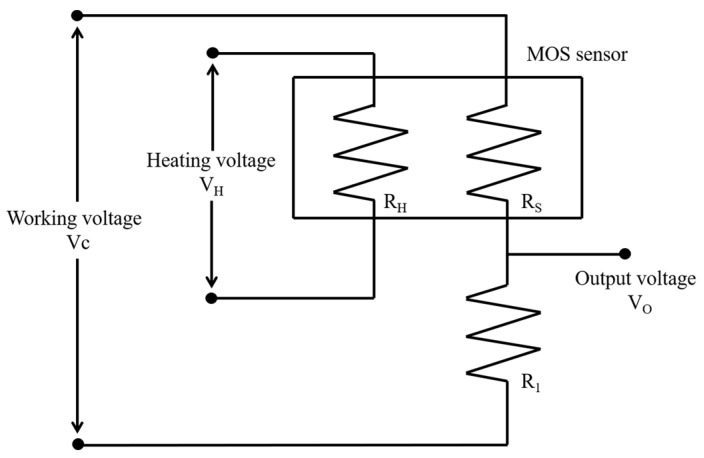
Typical signal output circuit of Au/ZnO-3.0% gas sensor in the acetone-detecting device.

To confirm the usability of the Au/ZnO-3.0% gas sensor, a compact, handheld, and cost-effective device, equipped with an early warning system for fast acetone detection, has been developed and produced. As a semiconductor metal oxide gas sensor, its typical signal output circuit is shown in Figure 7. R_H_ is a heating resistor that heats the Au/ZnO-3.0% gas sensor to bring it to its operating temperature. R_S_ is the resistance of the Au/ZnO-3.0% gas sensor, which is affected by the external environment. While the sensor comes into contact with the target gas, the resistance of the R_S_ resistor will change. Its resistance can also be affected by other factors, such as temperature and humidity. R_1_ is a pull-down resistor, which together with the gas sensor resistance affects the output voltage V_O_. When the value of R_S_/R_1_ increases or decreases, the corresponding output voltage V_O_ increases or decreases accordingly.

**Figure 8:**
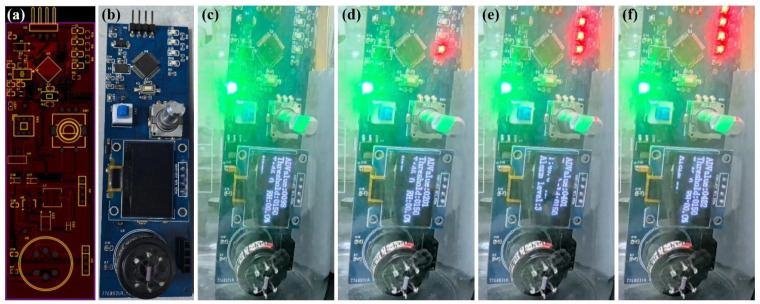
(**a**,**b**) Acetone-detecting device design; and (**c**–**f**) its display in acetone-detecting process.

Subsequently, a fully integrated device featuring an early warning system for acetone detection was developed and fabricated (Figure 8). The PCB design diagram of the device is shown in Figure 8a, which includes a main control chip module, a signal acquisition module, an encoder module, and an alarm module. The functions of each part are as follows:

(1) Main control chip module. The STM32F103C8T6 has been chosen as the primary control component to process and operate the collected data, and control and manage the device.
(2) Signal acquisition module. To gather the resistance signal from the Au/ZnO-3% gas sensor, the analog signal is converted into a digital signal, and the signal is transmitted to the main control chip.
(3) Encoder module. This adjusts the device alarm threshold to meet different environmental requirements.
(4) Alarm module. The LED signal light determines whether acetone gas is present via a threshold and increases the alarm level based on the gas concentration.

The size of the device is 10 cm × 3 cm, and its portability makes the device flexible in different scenarios. Au/ZnO-3.0% was chosen as the gas detection sensor. An image of the fabricated gas sensing device is shown in Figure S4. During the acetone detection process, the sensor collects the resistance signal and transmits the analog-to-digital converted signal to the primary control component. The signal is compared with the PWM encoder setting threshold (150, 250, 350, and 450 correspond to 30 ppm, 60 ppm, 90 ppm, and 120 ppm, respectively), and if the preset threshold is exceeded, the red alarm indicators light up. The alarm level of the device will continuously increase, along with the growth of the concentration of acetone (Figure 8c–f and Video S1). The OLED screen intuitively presents the signal sampling value, the preset threshold value, and the alarm level, which facilitates rapid data acquisition. Therefore, this device equipped with the Au/ZnO-3.0% sensor has efficient applications in the detection of acetone gas.

## 5. Conclusions

In summary, an efficient acetone gas sensor based on Au-modified ZnO porous nanofoam (Au/ZnO) was designed by PVP-blowing followed by a chemical calcination method. Combined XRD, SEM, TEM, and XPS characterizations with gas sensitivity tests investigated the effect of Au loading amount on acetone response for porous ZnO. The porous network nanostructure and electron spillover effect synergistically enhanced the gas-sensitive performances of the Au/ZnO sensor. The Au/ZnO sensor prepared by an Au loading ratio of 3.0% achieved a 100 ppm acetone gas response of 20.02 at the optimum working temperature of 275 °C. In addition, a portable electronic device integrating with the Au/ZnO-3.0% gas sensor achieved the function of detecting and alarming toxic acetone gas. This work provides a cost-effective strategy and device to detect acetone, which is of great significance in monitoring other toxic gas emissions.

## Data Availability

All relevant data are within the manuscript and its additional files.

## Supplementary Materials

The following supporting information can be downloaded at: https://www.mdpi.com/article/10.3390/s24248100/s1, Video S1: Acetone-detecting process by device; Figure S1: The cross-sectional SEM image of the Au/ZnO-3.0% coated on ceramic tube; Figure S2: The resistances in air at various working temperatures of the gas sensors; Figure S3: The response/recovery time of sensors to 100 ppm acetone at 275 °C. (a) ZnO, (b) Au/ZnO-0.5%, (c) Au/ZnO-1%, and (d) Au/ZnO-5%; Figure S4: The image of the fabricated gas sensing device; Table S1: Comparison of the acetone-sensing performance of the fabricated Au/ZnO porous nanofoam than that of sensors reported in literature.
