# Supplementary material for: Gas Sensor for Efficient Acetone Detection and Application Based on Au-Modified ZnO Porous Nanofoam
Source: Sensors (Basel). 2024 Dec 19;24(24):8100. doi: 10.3390/s24248100 (PMC11679938; doi:10.3390/s24248100)
Supplement: Supplementary file 1 [file sensors-24-08100-s001.zip › Supporting Information.pdf]

Supporting Information

Gas Sensor for Efficient Acetone Detection and Application Based on Au-Modified ZnO Porous Nanofoam

Zhenchao Sun [1,†], Shanfu Sun [1,*,†], Xidong Hao [1], Yinglin Wang [1], Caili Gong [2] and Pengfei Cheng [1,*]

[1] School of Aerospace Science and Technology, Xidian University, Xi'an 710126, China
[2] School of Electronic Information Engineering, Inner Mongolia University, Hohhot 010021, China
* Correspondence: sunshanfu@xidian.edu.cn (S.S.); pfcheng@xidian.edu.cn (P.C.)
† These authors contributed equally to this work.

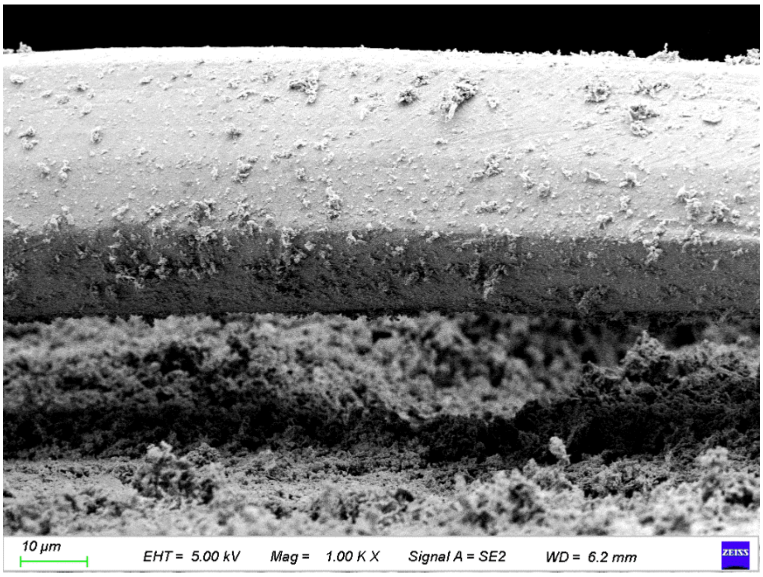

Figure S1. The cross-sectional SEM image of the Au/ZnO-3.0% coated on ceramic tube.

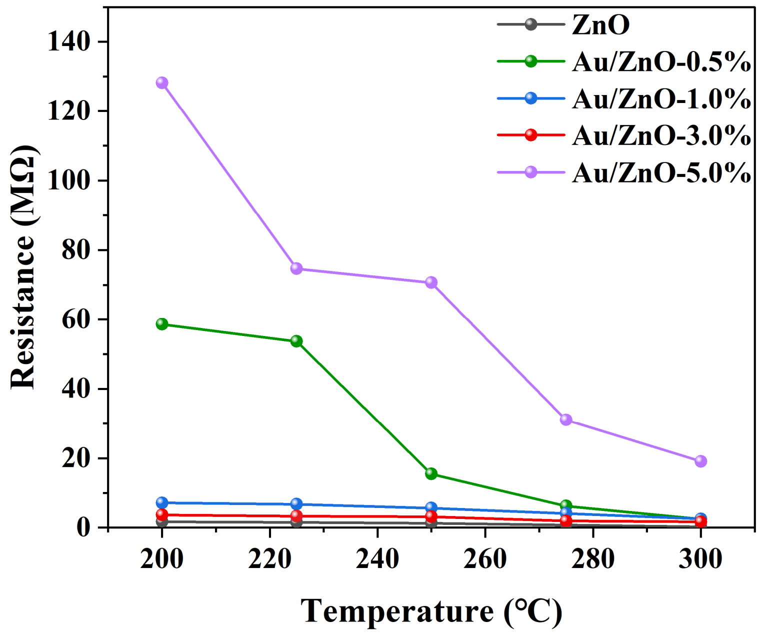

Figure S2. The resistances in air at various working temperatures of the gas sensors.

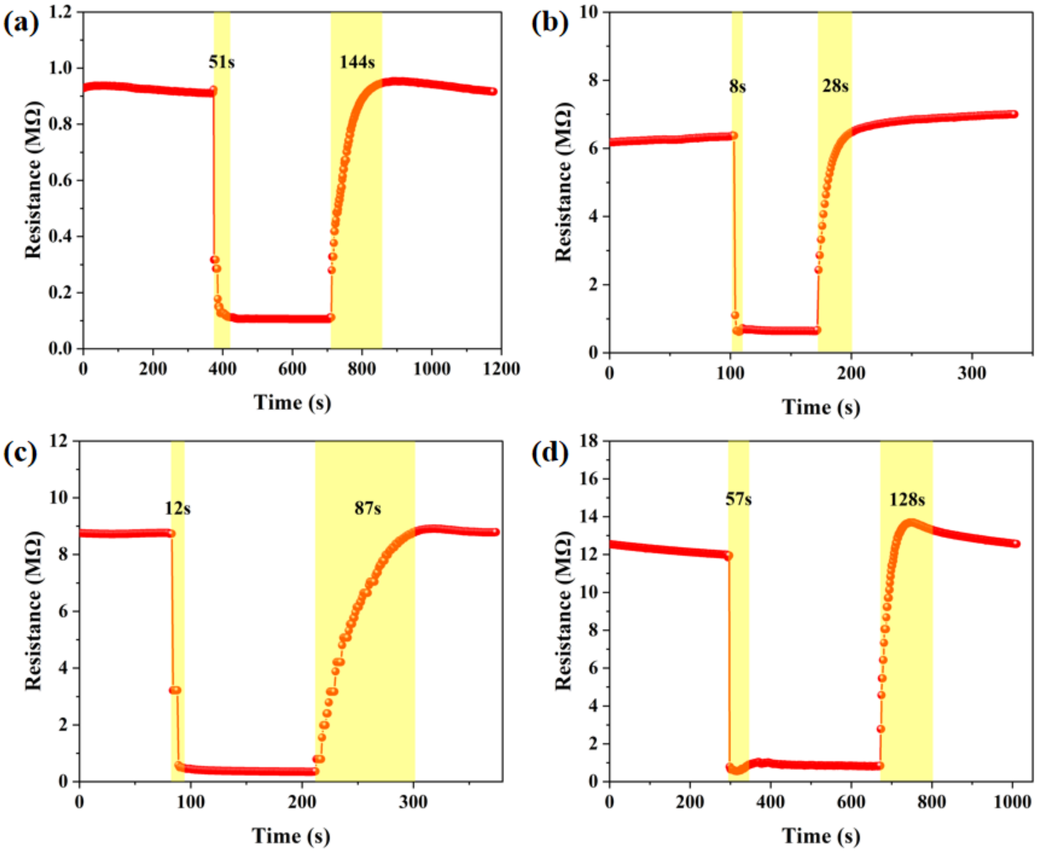

Figure S3. The response/recovery time of sensors to 100 ppm acetone at 275 °C. (a) ZnO, (b) Au/ZnO-0.5%, (c) Au/ZnO-1%, and (d) Au/ZnO-5%.

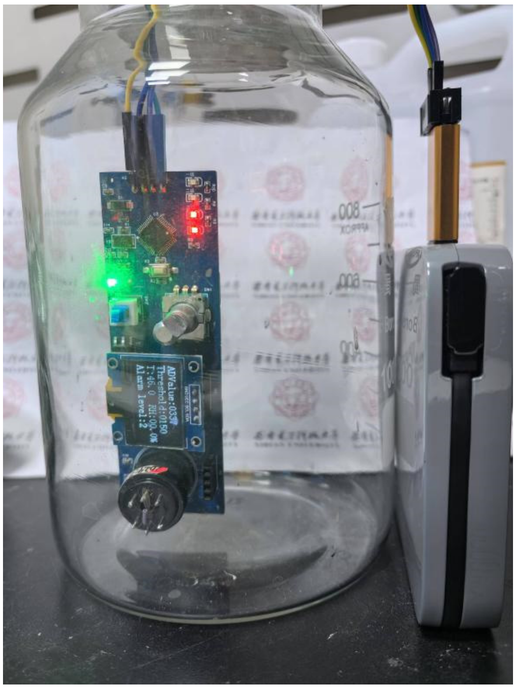

Figure S4. The image of the fabricated gas sensing device.

Table S1. Comparison of the acetone-sensing performance of the fabricated Au/ZnO porous nanofoam than that of sensors reported in literature.

Sensitive material	Optimum working temperature (°C)	Response value (Acetone concentration)	Reference
Au/ZnO porous nanofoam	275	20.02 (100 ppm)	This work
Flower-Like TiO_2	330	33.72 (250 ppm)	*Nanoscale Research Letters* 17 (2022) 82
SnO_2/Pd-NiO nanowires	450	14.88 (500 ppm)	*RSC advances* 14 (2024) 12438-12448
Au-loaded Co_3O_4	190	14.5 (100 ppm)	*Sensors and Actuators B: Chemical* 344 (2021) 130182
Pt-loaded Co_3O_4/$CoMoO_4$	250	24.12 (20 ppm)	*Physica E: Low-dimensional Systems and Nanostructures* 164 (2024) 116042.
Nb-doped CeO_2 nanofibers	240	23.27 (100 ppm)	*Applied Surface Science* 602 (2022) 154303
Ru/NiO-0.5% flower-like microspheres	200	12.6 (100 ppm)	*Sensors and Actuators B: Chemical* 313 (2020) 127965
Hollow CuO/Cu_2O octahedrons	350	3.52 (50 ppm)	*Sensors and Actuators B: Chemical* 423 (2025) 136783
C-d-mFe_2O_3 NRs	225	5.2 (2.5 ppm)	*Journal of Colloid and Interface Science* 622 (2022) 156-168
ZnO/$ZnFe_2O_4$ microspheres	250	94 (100 ppm)	*ACS Applied Nano Materials* 7 (2024) 16066-16074
Co_3O_4-decorated porous TiO_2 nanofibers	250	71 (100 ppm)	*Journal of Alloys and Compounds* 919 (2022) 165875
